# Mutations in SARS-CoV-2 variant nsp6 enhance type-I interferon antagonism

**DOI:** 10.1080/22221751.2023.2209208

**Published:** 2023-05-14

**Authors:** Cody J. Bills, Hongjie Xia, John Yun-Chung Chen, Jason Yeung, Birte K. Kalveram, David Walker, Xuping Xie, Pei-Yong Shi

**Affiliations:** a Department of Biochemistry and Molecular Biology, University of Texas Medical Branch, Galveston, TX, USA; b Department of Microbiology and Immunology, University of Texas Medical Branch, Galveston, TX, USA; c Department of Pathology, University of Texas Medical Branch, Galveston, TX, USA; d Center for Biodefense and Emerging Infectious Diseases, University of Texas Medical Branch, Galveston, TX, USA; e Sealy Institute for Drug Discovery, University of Texas Medical Branch, Galveston, TX, USA; f Institute for Human Infection and Immunity, University of Texas Medical Branch, Galveston, TX, USA; g Institute for Translational Sciences, University of Texas Medical Branch, Galveston, TX, USA; h Sealy Institute for Vaccine Sciences, University of Texas Medical Branch, Galveston, TX, USA; i Sealy Center for Structural Biology & Molecular Biophysics, University of Texas Medical Branch, Galveston, TX, USA

**Keywords:** SARS-CoV-2, variants, nsp6, interferon, cytokine storm

## Abstract

Severe acute respiratory syndrome coronavirus 2 (SARS-CoV-2) continues to evolve after its emergence. Given its importance in viral infection and vaccine development, mutations in the viral Spike gene have been studied extensively; however, the impact of mutations outside the Spike gene are poorly understood. Here, we report that a triple deletion (ΔSGF or ΔLSG) in nonstructural protein 6 (nsp6) independently acquired in Alpha and Omicron sublineages of SARS-CoV-2 augments nsp6-mediated antagonism of type-I interferon (IFN-I) signaling. Specifically, these triple deletions enhance the ability of mutant nsp6 to suppress phosphorylation of STAT1 and STAT2. A parental SARS-CoV-2 USA-WA1/2020 strain containing the nsp6 ΔSGF deletion (ΔSGF-WA1) shows reduced susceptibility to IFN-I treatment *in vitro*, outcompetes the parental strain in human primary airway cultures, and increases virulence in mice; however, the ΔSGF-WA1 virus is less virulent than the Alpha variant (which has the nsp6 ΔSGF deletion and additional mutations in other genes). Analyses of host responses from ΔSGF-WA1-infected mice and primary airway cultures reveal activation of pathways indicative of a cytokine storm. These results provide evidence that mutations outside the Spike protein affect virus-host interactions and may alter pathogenesis of SARS-CoV-2 variants in humans.

## Introduction

Severe acute respiratory syndrome coronavirus 2 (SARS-CoV-2) variants continue to emerge three years after SARS-CoV-2 was initially identified [1–4]. Due to the importance of the viral Spike protein in transmission and immunogenicity, COVID-19 vaccines utilize the Spike protein as the primary antigen [5–9]. Less attention has been devoted to studying the impact of variant mutations in nonstructural proteins (nsps) and accessory proteins and their roles in viral replication and pathogenesis [9]. Recent studies have reported the importance of non-spike mutations in promoting viral replication and antagonizing innate immune responses; for example, mutations in ORF8 were associated with increased virulence and antagonism of type-I interferon (IFN-I) pathways, exemplifying the need to understand the impact of non-spike mutations [10–18].

Two-thirds of the SARS-CoV-2 genome encodes two polypeptides (pp1a and pp1ab) that are cleaved into 16 nsps to form the replication complex [19,20]. The remaining third of the genome encodes 7 accessory and 4 structural proteins [19]. Many SARS-CoV-2 nsps and accessory proteins antagonize the interferon type I (IFN-I) response [21–25]. Nsp6 specifically inhibits both IFN-I induction and signaling pathways *in vitro* [21]. The Alpha variant of SARS-CoV-2, which was first identified in late 2020 in the United Kingdom and circulated globally, was reported to be less susceptible to treatment with IFN-I and IFN-III compared to the ancestral strain [26]. Based on these findings, we hypothesized that mutations in Alpha nsp6 would contribute to increased IFN-I resistance in the Alpha variant and other emerging variants of SARS-CoV-2. Here, we demonstrate that a convergent deletion in the nsp6 genes of Alpha and Omicron variants of SARS-CoV-2 confers a fitness advantage through enhanced antagonism of IFN-I signaling.

## Methods

### Ethics statement

Mouse studies were performed in accordance with the Care and Use of Laboratory Animals of the University of Texas Medical Branch (UTMB). The protocol (IACUC#: 2103023) received approval from the Institutional Animal Care and Use Committee (IACUC) at UTMB. Animals were anesthetized using isoflurane prior to operations to minimize animal suffering. Infections were performed in ABSL-3 facilities at UTMB by trained personnel.

### Cell culture and animal care

African green monkey kidney epithelial cells expressing human TMPRSS2 (Vero E6-TMPRSS2; purchased from SEKISUI XenoTech, LLC, Kansas City, KS) and human epithelial kidney cells (HEK293T; purchased from ATCC, Bethesda, MD) cells were cultured in high-glucose Dulbecco’s modified Eagle’s medium (DMEM; Gibco/Thermo Fisher, Waltham, MA, USA) supplemented with 10% fetal bovine serum (FBS; Hyclone Laboratories, South Logan, UT) and 1% penicillin/streptomycin (P/S; Gibco). All culture media and antibiotics were purchased from ThermoFisher Scientific (Waltham, MA). Primary human airway epithelial (HAE) cells and culture medium for HAE cells were purchased from MatTek Life Science (Ashland, MA, USA). All cell lines were maintained at 37°C with 5% CO_2_ and tested negative for mycoplasma contamination. Female K18-hACE2 c57BL/6J (strain: 2B6.Cg-Tg(K18-ACE2)Primn/J) mice aged 8–10 weeks were purchased from Jackson Laboratory (Bar Harbor, ME) and housed in ABSL-3 facilities at UTMB. Animals were randomized and housed in groups of <5 mice per cage and fed standard chow. The ABSL-3 rooms were maintained between 68–74°F with 30%−60% humidity. Lights maintained day/night cycles of 12 h intervals. Animals were allowed 3–4 days to acclimate before virus challenge.

### Constructing SARS-CoV-2 WA1-ΔSGF

The stock of SARS-CoV-2 strain 2019-nCoV/USA_WA1/2020 was isolated from the first COVID-19 patient diagnosed in the U.S. and provided to the World Reference Center for Emerging Viruses and Arboviruses (WRCEVA) at the University of Texas, Medical Branch (UTMB, Galveston, TX, USA). An infectious clone of the Alpha variant (GISAID: EPI_ISL_999340) was previously constructed [27]. SARS-CoV-2 infectious clones were constructed as previously described from a cDNA clone of USA-WA1/2020 and generated in Vero E6-TMPRSS2 cells [28,29]. All work following electroporation was performed in a biosafety level 3 (BSL-3) laboratory.

### IFN-α treatment of SARS-CoV-2

Vero E6-TMPRSS2 cells were seeded in 24-well plates (2 × 10^5^ cells/well) and incubated for at least 5 h. Cells were pre-treated for 16–18 h with IFN-α (Millipore, Darmstadt, Germany) diluted in 10% FBS DMEM. Cells were washed with DPBS and then infected at MOI 0.02 with 0.2 mL WA1 or ΔSGF-WA1 diluted in 2% FBS DMEM medium and incubated for 1 h at 37°C with 5% CO_2_. Inoculum was removed and cells were washed with DPBS then fresh IFN-α diluted in 2% FBS DMEM medium was added. After 48 h incubation, supernatants were harvested for plaque assays and RT-qPCR.

### Plaque assays and reverse transcription quantitative PCR

Infectious virus from experiments was quantified using plaque assays performed as previously described using Vero E6-TMPRSS2 cells [30].

To quantify viral RNA *in vitro*, 0.2 mL infected culture supernatants were harvested 48 h post-infection and added to 4 volumes of Trizol LS Reagent (Thermo Fisher Scientific). RNA was purified using Direct-zol RNA Miniprep Plus Kits (Zymo, Irvine, CA) according to the manufacturer’s instructions, eluted in 50 μL nuclease-free water, then amplified using iTaq™ Universal SYBR® Green One-Step Kit (Bio-Rad) with QuantStudio™ 7 Flex system (ThermoFisher Scientific). The Ct values of the N gene were normalized to the Ct values of the M-GAPDH for mouse lung tissues or HuGAPDH for HAE cells. Table S1 summarizes the sequences for primer sets.

### Mouse challenge

To compare variants *in vivo*, 8-week-old female K18-hACE2 mice were anesthetized with isoflurane and then challenged intranasally with 50 μL (25 μL per nostril) inoculum of WA1 or ΔSGF-WA1 virus normalized to 10^3^ PFU/dose. For day 2, ten mice were in each infection group; for days 4 and 6, 14 mice were included in each group; four mice were mock infected, totalling 80 mice (Figure 2D-F). For survival experiments, ten mice were included in each group, totalling 40 mice (Figure 2G,2H). After inoculation, the K18-hACE2 mice were weighed daily and evaluated and scored based on visible indicators until the termination of the experiment. At the end of the experiment, mice were anesthetized and tissue samples were collected in 2 mL tubes containing PBS for plaque assays or TRIzol Reagent for RNA purification and stored at −80°C until use. Tissues were weighed and then processed by homogenizing with glass beads for 60 s at 6000 rpm using a MagNA Lyser (Roche Diagnostics) and centrifuged for 5 min at 10,000 rpm. Supernatants were transferred to fresh tubes for downstream analysis and then stored at −80°C. RNA was purified using the Direct-zol-96 MagBead RNA kit (Zymo) with a KingFisher Apex System (ThermoFisher Scientific).

### Histology

Left lungs from mice were harvested on days 4 and 6 and fixed in 10% buffered formalin solution for 7 days. The lung tissues were embedded in paraffin and cut into sections 5 μm thin to mount on slides, then stained with hematoxylin and eosin (H&E) on a SAUKRA VIP6 processor at the UTMB Histology Laboratory. Histology slides were scored by an independent histopathologist.

### Competition assays

HAE cells were infected with a mix of the WA1 or mutant virus with an equal MOI of 0.4 and samples were collected as previously described [30]. For analysis, RNA was purified from each sample and cDNA fragments corresponding to codon positions 72–147 of nsp6 were generated using the SuperScript™ IV One-Step RT–PCR System. See Supplementary Information for primer sets.

### IFN-I induction and signaling luciferase assays

Luciferase assays were performed as previously described [21,31], except plasmids were transfected into 1 × 10^5^ HEK293T cells using X-treme-GENE™ 360 (Roche, Mannheim, Germany) with a ratio of 1:1.

### SDS-PAGE and Western blots

Exogenous expression of viral proteins and endogenous proteins were detected using Western blots as previously described [21].

### Analysis of nCounter analysis system (NanoString) data

RNA was purified from mouse lung tissues as described above and concentrations were normalized to 20 ng/μL. The RNA was prepared and analyzed using the nCounter Pro Analysis System and the nSolver Analysis Software. All plots in Figure 3 were made using R version 4.1.2. An un-adjusted *p*-value cutoff of 0.05 was used to determine differentially expressed genes (DEGs) as adjusted *p*-values did not reach statistical significance. An additional log2 fold change cutoff of + or – 0.6 was used to label up- and down-regulated genes in volcano plots for each condition. The Venn diagram for differentially expressed genes across conditions was made using the “ggvenn” package version 0.1.9. The “biomaRt” package version 2.50.3 was used to convert between human and mouse gene names to detect overlapping DEGs. Ingenuity pathway analysis (version 84978992) core analysis was performed on data (gene name, un-adjusted *p*-value, and fold change) from each condition, evaluating based on expression fold changes. The Ingenuity Knowledge Base (Genes Only) was used as the reference set. Canonical pathway and upstream regulator data was derived from a comparison analysis including mouse day 2 & 4, and human airway epithelial day 2 groups. -log(*p*-values) and z-scores were used to generate bubble plots, using ascending *p*-value or descending -log(*p*-value) for ordering.

### Statistical analysis

Data are presented as means ± standard deviations. Statistical significance was performed using Student’s T-test, One-Way ANOVA, or a Log-rank Mantel–Cox test calculated with the software Prism 9 (GraphPad) version 9.5.0.

### Data availability

The data supporting these findings are available upon request from the corresponding authors.

## Results

### Deletions in the nsp6 105–108 region arose independently in SARS-CoV-2 variants

**Figure 1.**
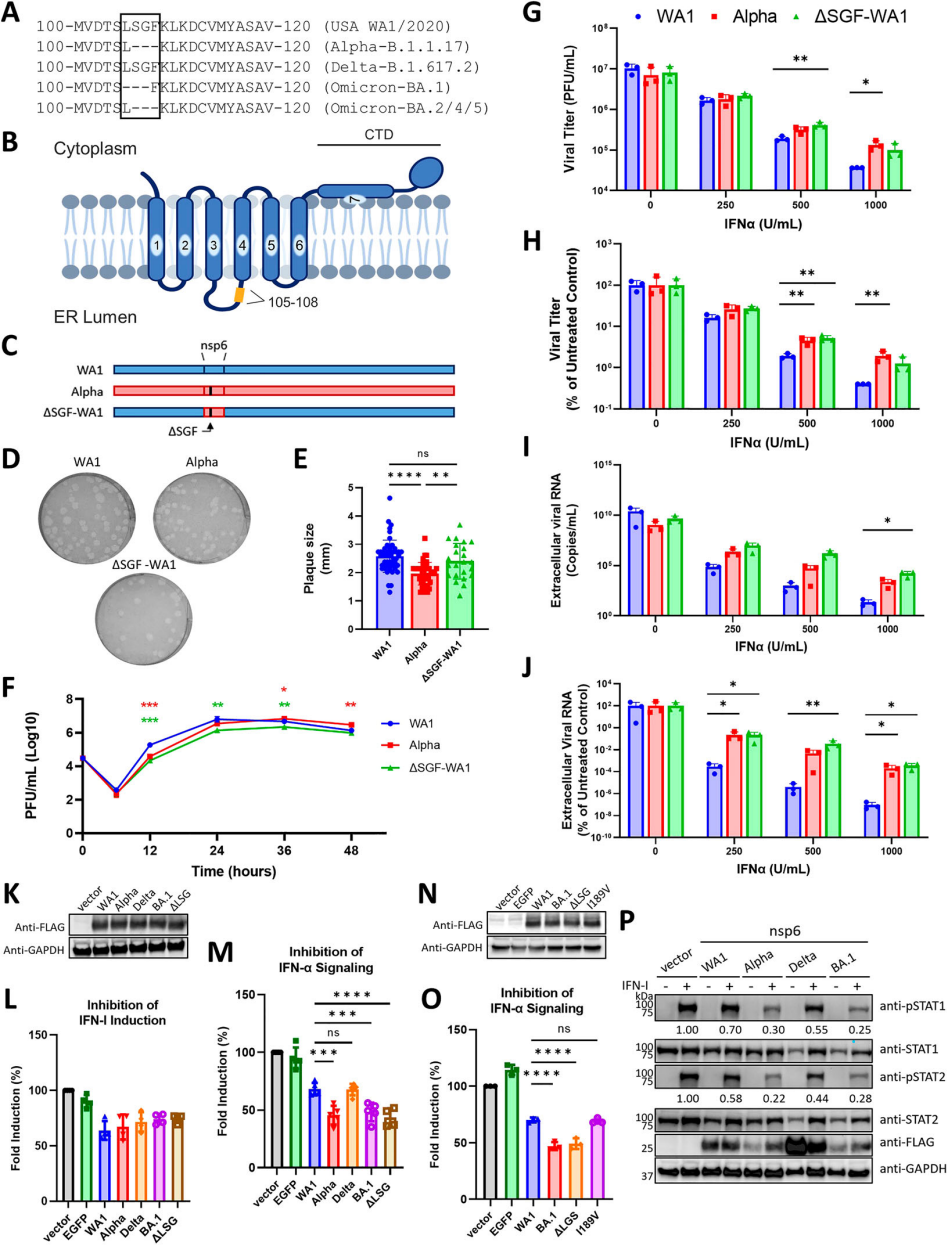
ΔSGF and ΔLSG enhance nsp6 suppression of IFN-α signaling. (A) Alignment of parental and variant nsp6 amino acid sequences of the region containing the independently acquired triple deletions. Delta nsp6 contains a unique V149A mutation and BA.1 nsp6 contains I189V in addition to ΔLSG. (B) Diagram of the predicted structure of nsp6 with enumerated transmembrane domains and the 105–108 region, where ΔSGF and ΔLSG occur, highlighted in yellow. (C) Schematic of the parental WA1, Alpha variant, and mutant ΔSGF-WA1 infectious clone generated by reverse genetics. (D) Representative images of plaque morphologies and (E) comparison of average plaque sizes. (F) Viral titers from a growth kinetics experiment where Vero E6-TMPRSS2 cells were infected at MOI 0.02 and supernatants were collected for plaque assays. (G) Raw viral titers from supernatants of Vero E6-TMPRSS2 cells infected with SARS-CoV-2 viruses at MOI 0.02 and treated with 2-fold serial dilutions of IFN-α. (H) Viral titers from (G) presented as a percent of untreated controls (0 IFN-α U/mL). (I) Extracellular viral RNA from supernatants of IFN-α treated quantified by RT-qPCR using a standard curve. (J) Extracellular viral RNA from (I) normalized to untreated controls (0 IFN-α U/mL). (G-J) Data are representative of three replicate experiments. (K) Western blot validating protein expression of nsp6 variant genes from pXJ plasmids in HEK293T cells. (L) IFN-α induction assay and (M) IFN-α signaling assay in HEK293T cells; values represent measured Firefly luciferase signals normalized to Renilla luciferase signals then normalized to the vector control; data are combined from at least three replicate experiments; no statistical differences were detected for (L). (N) Western blot demonstrating expression of nsp6 variant genes from pXJ plasmids in HEK293T. (O) IFN-α signaling assay in HEK293T cells presented as in (M). (P) Representative Western blot from three replicate experiments measuring levels of phosphorylated STAT1 or STAT2 (pSTAT1 or pSTAT2) in cells transfected with respective variant nsp6 genes and treated with IFN-α; values represent fold change of pSTAT1 or pSTAT2 for each variant nsp6 gene relative to vector control and normalized to GAPDH. Significance was determined using One-Way ANOVA with ns (not significant), *p* ≤ 0.05 (*), *p* ≤ 0.01 (**), and *p* ≤ 0.001 (***). Diagram (B) was created using BioRender. Additional figures created using GraphPad Prism 9.

SARS-CoV-2 WA1 strain was isolated from the first imported COVID-19 patient in the United States, and is the standard strain used for SARS-CoV-2 experiments [1,2]. Alignment of nsp6 sequences reveals a convergent deletion of amino acids 106–108 (ΔSGF) in Alpha and Omicron sublineages (BA.2, BA.4, BA.5; Figure 1A). Notably, Omicron BA.1 also contains a deletion that is shifted to 105–107 (ΔLSG), as well as a unique I189V mutation. Delta nsp6 contains only a unique V149A change, suggesting that ΔSGF and ΔLSG emerged independently in the Alpha and Omicron sublineages and such deletions may have biological functions and consequences. Sequence analysis showed that ΔSGF and ΔLSG were present in 42% and 19% of the ∼15 million GISAID sequences, respectively. ΔSGF was prevalent in at least 95% of contemporary sequences, including XBB.1.5, CH.1.1, and BQ.1.1, whereas ΔLSG accounted for <0.5% (Table S2) [32].

The nsp6 structure is predicted to form 7 transmembrane domains. The last domain is amphipathic and may associate with, but does not traverse, the membrane. Thus, both the N- and C-termini lie in the cytoplasm (Figure 1B) [12,33]. The 105–108 region, where ΔSGF and ΔLSG deletions occur, lies in an unstructured lumenal loop between transmembrane domains 3 and 4 (Figure 1B), a region thought to play a role in nsp6-mediated ER zippering [12,33].

### ΔSGF-WA1 SARS-CoV-2 replicates like the Alpha variant

To investigate the biological function of nsp6 mutations, we introduced ΔSGF into WA1 SARS-CoV-2 (ΔSGF-WA1) using a reverse genetics system described previously (Figure 1C) [28,29]. We first sought to compare the replication of ΔSGF-WA1 to WA1 and Alpha in IFN-I production-deficient Vero E6-TMPRSS2 cells. Mutant ΔSGF-WA1 produced plaques similar to WA1, but slightly larger than Alpha (Figure 1D, 1E). In a replication kinetics experiment, WA1 titers peaked at 24 h, while Alpha and ΔSGF-WA1 peaked at 36 h and maintained peak titers for slightly longer than WA1 (Figure 1F). Thus, ΔSGF-WA1 replication appears to resemble Alpha SARS-CoV-2 more than WA1.

### ΔSGF-WA1 mutant is less susceptible to IFN-α treatment

We next examined whether ΔSGF-WA1 showed reduced susceptibility to IFN-α treatment as observed with the Alpha variant in previous studies [26]. Vero E6-TMPRSS2 cells were pre-treated with IFN-α and subsequently infected with WA1, Alpha, or ΔSGF-WA1 SARS-CoV-2. Both ΔSGF-WA1 and Alpha produced significantly higher titers than WA1 when treated with 500 or 1000 U/mL of IFN-α (Figure 1G). After normalizing virus titers to the untreated controls, ΔSGF-WA1 replicated to higher levels than WA1 at 500 U/mL of IFN-α (Figure 1H). Raw extracellular viral RNA levels were higher for ΔSGF-WA1 compared to WA1 when treated with 1000 U/mL of IFN-α (Figure 1I), and normalized extracellular ΔSGF-WA1 RNA was consistently higher than WA1 at every IFN-α concentration (Figure 1J). The overall data suggest that ΔSGF reduced IFN-α sensitivity of the Alpha variant.

### Deletions in the 105–108 region of nsp6 augment antagonism of IFN-α signaling

To understand how variant nsp6 mutations contribute to IFN-α resistance, we performed an IFN-I induction assay as previously described [21,31]. Briefly, we cloned nsp6 genes from WA1, Alpha (ΔSGF), Delta (V149A), and Omicron BA.1 (ΔLSG + I189V) variants into a pXJ expression plasmid with a C-terminal FLAG tag (Figure 1K). To determine whether BA.1 ΔLSG has the same effect as Alpha ΔSGF, we also cloned a ΔLSG nsp6 plasmid. To evaluate IFN-I production, the nsp6 gene plasmids were co-transfected into HEK293T cells with (i) a plasmid encoding a firefly luciferase gene controlled by the IFN-β promoter, (ii) a plasmid containing the RIG-I gene with a CARD domain, which renders the expressed RIG-I constitutively active, and (iii) a plasmid expressing Renilla luciferase to normalize transfection efficiencies. An empty pXJ plasmid and pXJ-EGFP were transfected as controls. At 24 h post-transfection, luciferase signals were measured to quantify the effect of nsp6 protein on IFN-β promoter activity. As expected, WA1 nsp6 reduced luciferase signals by about 36% (Figure 1 K, 1L). The luciferase signals from the Alpha (ΔSGF), Delta (V149A), and BA.1 (ΔLSG + I189V or ΔLSG) nsp6-expressing cells were not significantly different from the WA1 nsp6-expressing cells (Figure 1L). The results suggest that nsp6 mutations do not affect nsp6 antagonism of IFN-I induction.

Next, we tested whether the nsp6 mutations modulate IFN-I signaling. Using a similar luciferase assay, we co-transfected HEK293T cells with three plasmids: (i) an nsp6-expressing plasmid, (ii) a firefly luciferase plasmid regulated by the ISRE promoter, and (iii) a control Renilla luciferase plasmid. The transfected cells were treated with 250 U/mL of IFN-α to determine whether variant nsp6 proteins repress ISRE promoter activity, as measured by the luciferase signal. As expected [21], the WA1 nsp6 reduced luciferase signals by 32%; Alpha (ΔSGF), BA.1 (ΔLSG + I189V), and BA.1 (ΔLSG) nep6 reduced luciferase signals by 55%, 52%, and 56%, respectively (Figure 1M). Repression of IFN-α signaling by Delta (V149A) nsp6 was not different from WA1 (Figure 1M). These results suggest that both ΔSGF and ΔLSG mutations enhance nsp6’s antagonism of IFN-α signaling, which may drive IFN-α resistance in the Alpha variant and likely Omicron sublineages.

To validate whether ΔLSG found in BA.1 nsp6 indeed contributes to enhanced antagonism of IFN-α signaling, we constructed an additional plasmid expressing BA.1 nsp6 that restored the LSG sequence but retained the I189V mutation [i.e. BA.1 (I189V) nsp6; Figure 1N]and repeated the IFN-α signaling assay. As expected, inhibition of IFN-α signaling by BA.1 (I189V) nsp6 was comparable to WA1 nsp6, while IFN-α signaling was reduced significantly more by BA.1 (ΔLSG) nsp6 (Figure 1O). These data confirm the results above and demonstrate that ΔLSG in the 105–108 region of nsp6 enhances inhibition of IFN-α signaling.

### ΔSGF and ΔLSG augment the suppression of STAT1 and STAT2 phosphorylation

We previously showed that nsp6 represses the IFN-I signaling pathway by blocking phosphorylation of STAT1 and STAT2 [21]. So, we hypothesized that Alpha and Omicron nsp6 would more potently block STAT1 and STAT2 phosphorylation. To test this hypothesis, we transfected HEK293T cells with variant nsp6 plasmids or a vector control, treated the cells with IFN-α, and analyzed phosphorylation of STAT1 and STAT2 using Western blotting. WA1 nsp6 reduced STAT1 and STAT2 phosphorylation by 30% and 42%, respectively (Figure 1P). Alpha (ΔSGF), Delta (V149A), and BA.1 (ΔLSG + I189V) nsp6 further reduced phosphorylation of both STAT1 by 70%, 45%, and 75%, respectively; and STAT2 by 78%, 56%, and 72% (Figure 1P). These results demonstrate that Alpha and Omicron nsp6 more efficiently reduce phosphorylation of STAT1 and STAT2 compared to controls; Delta, which lacks the 105–108 deletion, reduced STAT1 and STAT2 phosphorylation slightly more than WA1, but not to the same degree as Alpha or Omicron (Figure 1P). This might suggest that the unique V149A mutation in Delta nsp6 still enhances the blockage of STAT1 and STAT2 phosphorylation, but not as well as ΔSGF and ΔLSG. Altogether, these data demonstrate that ΔSGF and ΔLSG in the 105–108 region of nsp6 enhance its antagonism of IFN-α signaling by more efficiently suppressing phosphorylation of STAT1 and STAT2.

### ΔSGF-WA1 outcompetes WA1 in primary human airway epithelial cells

**Figure 2.**
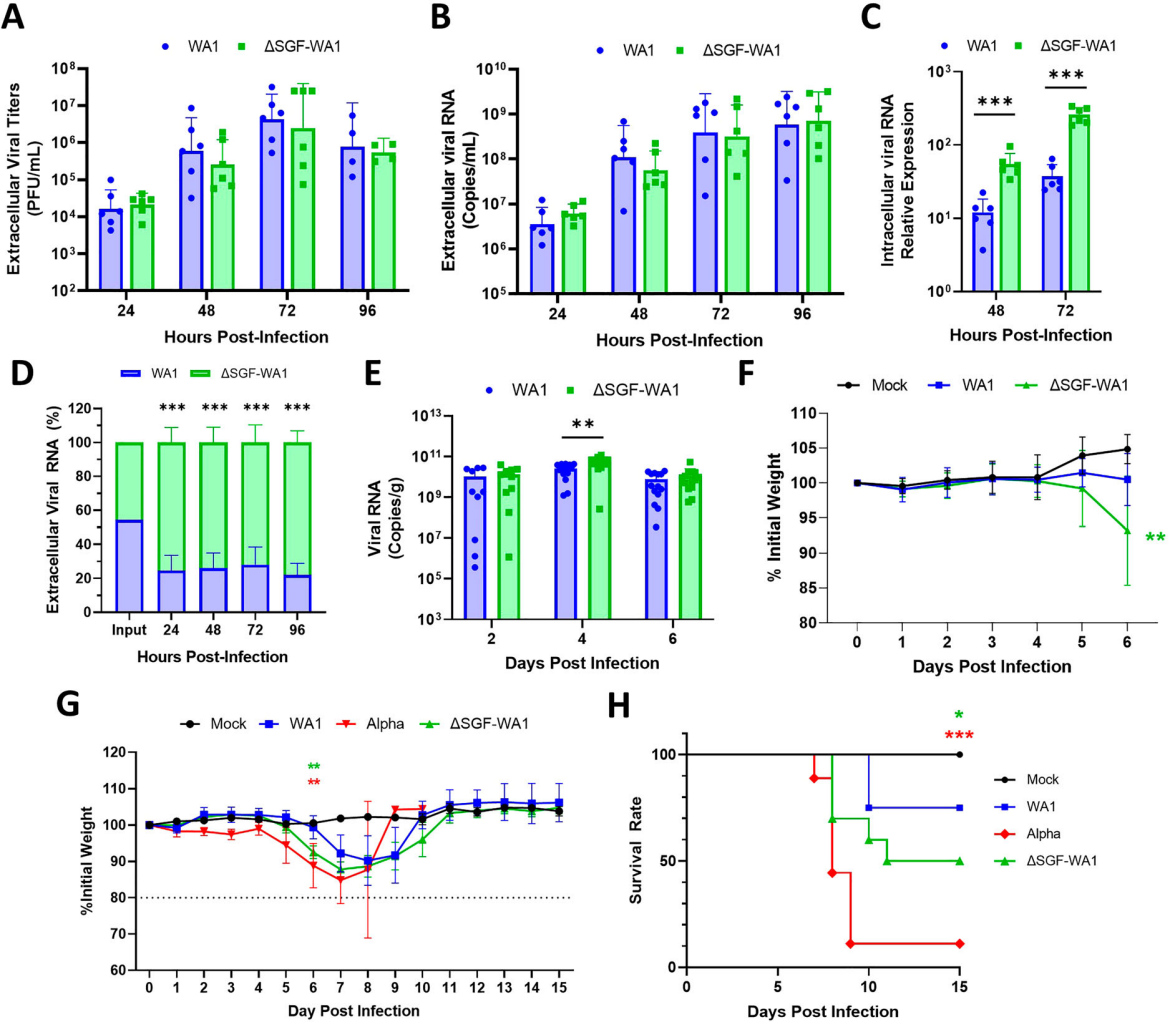
ΔSGF-WA1 outcompetes WA1 in HAE cells and augments disease severity in mice. (A) Extracellular viral titers and (B) levels of viral RNA over 96 h from supernatants of infected HAE cells at MOI 0.4 using (A) plaque assays and (B) RT-qPCR with a standard curve. (C) Levels of intracellular viral RNA from infected HAE cell lysates normalized to GAPDH. (D) Competition assay from the supernatants of HAE cells infected with equal MOI of WA1 and ΔSGF-WA1. Copy numbers of each virus were quantified using next-generation sequencing and are presented as percentages of the total number of viral copies. (E) Viral RNA measured by RT-qPCR from infected mouse lung tissues normalized to tissue weights measured by plaque assays. Samples were harvested on days 2 (10 mice per virus), 4 (14 mice per virus), and 6 (14 mice per virus; and 4 mock-infected mice). (F) Average weights of mice infected with WA1 SARS-CoV-2 or ΔSGF-WA1 measured daily. Significance is based on a comparison of ΔSGF-WA1 to WA1. (G) Average weights of infected mice and (H) survival curves over 15 days (10 mice per group). Mice were humanely euthanized if the weight dropped below 80% initial weight (dashed line). Significance was determined using Student’s T-test for each timepoint compared to WA1 or using a Log-rank Mantel-Cox test (H) with *p* ≤ 0.05 (*), *p* ≤ 0.01 (**), and *p* ≤ 0.001 (***).

To evaluate ΔSGF-WA1 replication in a more relevant cell line, we infected primary human airway epithelial (HAE) cells. After 96 h, ΔSGF-WA1 infectious titers and extracellular viral RNA were comparable to WA1 at each timepoint (Figure 2A, 2B). However, intracellular ΔSGF-WA1 viral RNA was significantly higher than WA1 after 48 and 72 hpi (Figure 2C). The higher levels of intracellular ΔSGF-WA1 RNA could result from improved antagonism of IFN-I responses; however, it’s unclear why ΔSGF-WA1 does not produce higher levels of extracellular viruses. Previous reports indicate that ΔSGF improves nsp6-mediated formation of replication organelles, providing better protection of replicating RNA from immune sensors and thus leading to higher levels of intracellular viral RNA [12,34–39]; however, the enhanced replication organelles may reduce the efficiency of virion assembly and/or release. Future experiments are needed to test these hypotheses.

To improve the sensitivity of viral fitness experiments, we performed a competition assay by infecting HAE cells with both WA1 and ΔSGF-WA1 with an equal MOI of 0.4. Next-generation sequencing was used to quantify the proportion of two viral RNA species. In contrast to the non-competition experimental results (Figure 2A, 2B), the competition assay showed that ΔSGF-WA1 outcompeted WA1 by 24 hpi (Figure 2D); the discrepancy is most likely caused by the difference in experimental sensitivity. The competition assay has been proven more sensitive to compare viral fitness due to the elimination of host-to-host variation [40–43].

### ΔSGF-WA1 SARS-CoV-2 is more virulent than WA1, but less than Alpha variant, in K18-hACE2 mice

To examine whether ΔSGF affects the *in vivo* virulence of SARS-CoV-2, we infected K18-hACE2 mice with 10^3^ PFU WA1 or ΔSGF-WA1, and quantified the lung viral loads at different days post-infection (dpi). ΔSGF-WA1 generated significantly more lung viral RNA than WA1 at 4 dpi (*p* = 0.0031) and a similar trend was observed at 2 and 6 dpi, but the differences were not statistically significant (*p* = 0.6455 and *p* = 0.3746, respectively; Figure 2E). Interestingly, mice infected with ΔSGF-WA1 lost significantly more weight by 6 dpi than WA1-infected mice (*p* = 0.0040; Figure 2F). However, histopathology analysis of the infected lungs revealed similar histopathology scores at 4 and 6 dpi (Fig. S1), suggesting that infections with WA1 or ΔSGF-WA1 develop similar severity of lung pathology.

To further analyze disease severity in ΔSGF-WA1-infected mice, we performed a 15-day experiment to compare weight loss and survival rates of mice intranasally inoculated with WA1, ΔSGF-WA1, or full-length Alpha variant. Both Alpha- (*p* = 0.0039) and ΔSGF-WA1-infected mice (*p* = 0.0084) began losing weight on day 5 and, by day 6, had already lost significantly more weight than WA1-infected mice (Figure 2G). Mice infected with ΔSGF-WA1 did not fully recover until approximately day 11, while WA1-infected mice recovered by day 10, representing a disease period of 6 and 4 days for ΔSGF-WA1- and WA1-infected mice, respectively (Figure 2G). Alpha-infected mice experienced a greater average weight loss (15.2%) than ΔSGF-WA1- (12.2%) and WA1-infected mice (7.8%; Figure 2G). An accurate estimate of the disease period for the Alpha variant cannot be determined because, by day 9, the survival rate for Alpha-infected mice was only 10% (Figure 2H). Mice infected with WA1 or ΔSGF-WA1 had a 75% or 50% survival rate, respectively (Figure 2H). Together, these results indicate that ΔSGF-WA1 is more virulent in mice than WA1, but less virulent than Alpha.

### Upregulation of cytokine storm in ΔSGF-WA1-infected mice and HAE cells

**Figure 3.**
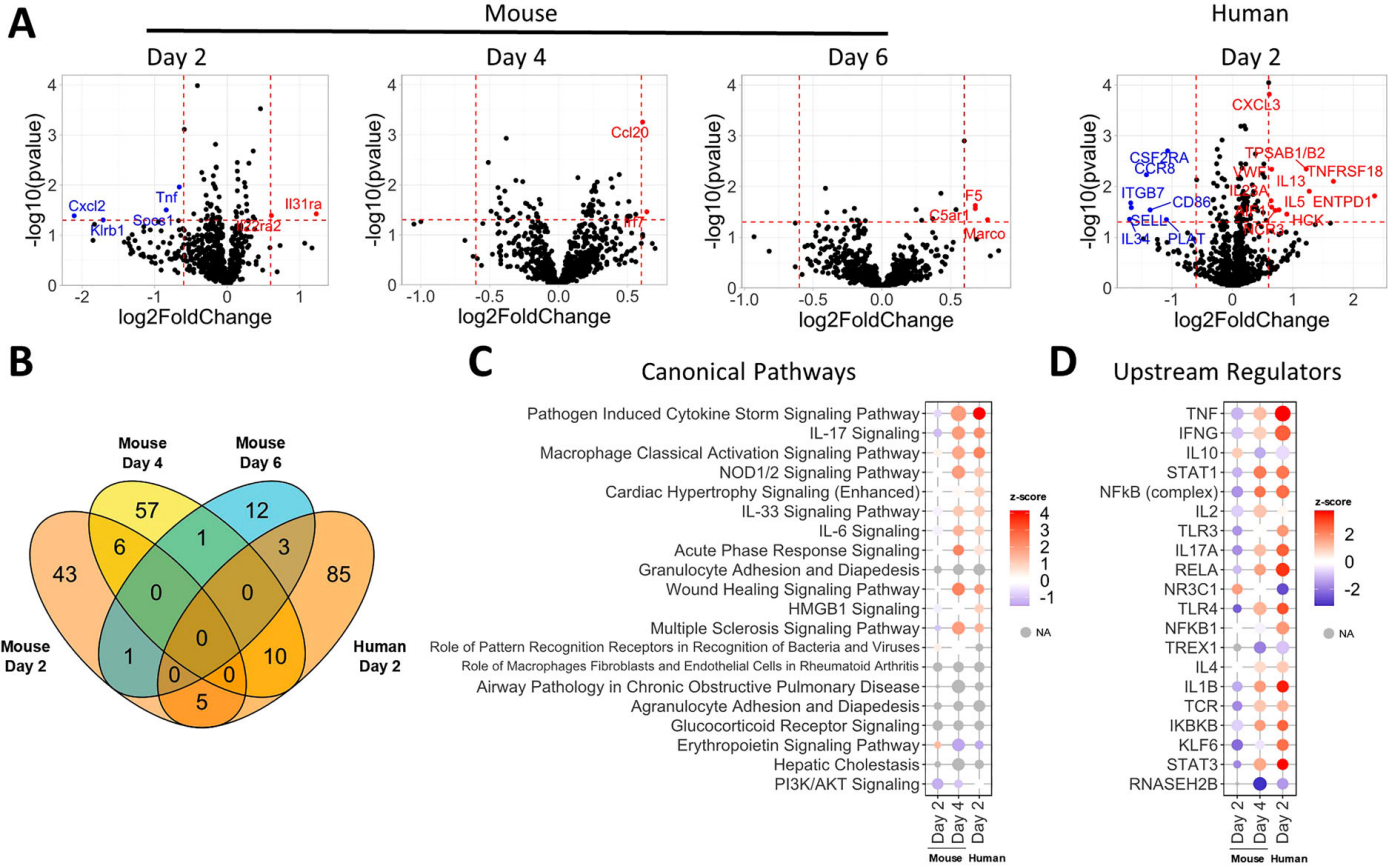
ΔSGF alters host responses causing extensive cytokine expression. (A) Volcano plots from nCounter Analysis for mouse whole lung specimens and human airway epithelial cultures (HAE) at specified days post infection. All comparisons are between nsp6 mutant and WT. Horizontal dotted line corresponds to a *p*-value cutoff of 0.05 and vertical lines correspond to −0.6 and 0.6 log2(fold change). (B) Venn diagram of differentially expressed genes between four conditions (Day 2 mouse, Day 4 mouse, Day 6 mouse, and Day 2 HAE). (C) Bubble plot of the top 20 statistically significant canonical pathways by ascending *p*-value from comparison analysis in Ingenuity Pathway Analysis. Dot size corresponds to –log(*p*-value). Colour corresponds to activation z-score communicating the directionality (activation or inhibition) for that pathway. Grey indicates z-score values which could not be calculated. (D) Bubble plot of the top 20 statistically significant upstream regulators by ascending *p*-value from comparison analysis in Ingenuity Pathway Analysis.

To understand how nsp6 ΔSGF affects host responses to SARS-CoV-2 infection, we compared mRNA expression of 785 host genes from lung tissues of WA1- and ΔSGF-WA1-infected mice, as well as infected HAE cells, using the probe-based nCounter Analysis System. These analyses revealed a total of 43, 57, and 12 differentially expressed genes in ΔSGF-WA1-infected mouse lung tissues on 2, 4, and 6 dpi, respectively, and 85 differentially expressed genes in ΔSGF-WA1-infected HAE cells at 2 dpi, compared to WA1 infections (Figure 3A). Of these, 26 total genes were shared between different pairwise groups (Figure 3B). Ingenuity Pathway Analysis (IPA) identified “Pathogen Induced Cytokine Storm Signaling Pathway” as a significantly altered host pathway for three datasets that was initially downregulated 2 dpi in mouse tissues, then significantly upregulated 4 dpi in ΔSGF-WA1-infected mouse tissues and 2 dpi in HAE cells (Figure 3C). Additional pathways identified by IPA support this finding, namely, IL-17, NOD1/2, and IL-6 signaling pathways, which also contribute to cytokine storms, were significantly upregulated in ΔSGF-WA1-infected mice and HAE cells (Figure 3C). Furthermore, IPA identified various upstream regulators of pyroptosis and cytokine storms such as TNF, NF-kB, MAVS, and IL1B (Figure 3D). Pathways identified by IPA for mouse tissues harvested 6 dpi did not overlap with other timepoints and instead were involved in resolution of inflammation and repair (See Supplementary Materials). It should be noted that pathways and upstream regulators from day-2 mouse tissues reveal a distinct pattern from the other data sets, suggesting a dynamic and complex *in vivo* virus-host interaction network. STAT1, an important component of the IFN-I pathway, was identified as an upstream regulator that was upregulated by day 4 in mice and day 2 in HAE cells; the expression of other components of the IFN-I signaling pathway was not significantly altered. We suspect that the suppression of IFN-I signaling by ΔSGF-WA1 may delay initial host responses, allowing for the accumulation of viral RNA, and eventually triggering an overpowering immune response to suppress the viral load. These data suggest that ΔSGF-WA1 causes dysregulated inflammatory responses and cytokine storm in mice and HAE cells.

## Discussion

Our results showed that variant mutations in nsp6 alter the virulence of SARS-CoV-2 through enhanced antagonism of STAT1 and STAT2 phosphorylation and dysregulation of inflammatory cytokines. The mechanism by which nsp6 antagonizes IFN-α pathways is currently unclear. Based on the predicted topology of SARS-CoV-2 nsp6, the 105–108 region resides in the ER lumen. It is currently unclear how ΔSGF and ΔLSG affects protein–protein interactions with components of the IFN-α signaling pathway. Shortening the lumenal loop might impact the overall structure of nsp6, leading to altered protein–protein interactions on the cytoplasmic side. Proteomics studies have identified some host interactors, but none of them are related to the IFN-α signaling pathway [44]. Thus, it is important to study nsp6’s interactions with specific components of the IFN-α pathway. Alternatively, viral infections can induce ER stress responses to protect cells from apoptosis and allow continued viral replication [44,45]; activated ER stress may prevent maturation and presentation of the IFN-α receptor 1 (IFNAR1).

Coronavirus nsp6 localizes almost exclusively to the ER where, in collaboration with nsp3 and nsp4, nsp6 promotes the formation of double membrane vesicles (DMVs) that protect replicative viral RNA from host sensors [12, 34–39]. ΔSGF was reported to enhance nsp6-mediated ER zippering (bringing the ER membranes close together with barely visible lumen) for more efficient formation and organization of replication organelles [12]. In line with this theory, we show that mutant ΔSGF-WA1 produces higher levels of intracellular viral RNA and outcompetes the parental strain in HAE cells (Figure 2C, 2D); however, ΔSGF-WA1 produced similar levels of secreted virus particles and viral RNA as WA1 in HAE cells (Figure 2A, 2B). The discrepancy between the intracellular and extracellular viral RNA levels may be caused by a negative effect of nsp6 (ΔSGF) on virus assembly and/or release.

Mice infected with ΔSGF-WA1 experienced more severe disease that resulted in earlier weight loss, a longer recovery period, and lower survival rates compared to WA1-infected mice (Figure 2G, 2H). IPA analysis of data from the nCounter Analysis System provides evidence that more severe disease and higher mortality in ΔSGF-WA1-infected mice are likely due to pathogen induced cytokine storm. Many of the pathways and upstream regulators are unaffected or even downregulated at 2 dpi but then upregulated by 4 dpi. This may be because ΔSGF-WA1 more efficiently suppresses initial host responses thereby allowing for increased replication of viral RNA, resulting in an overwhelming upregulation of cytokine responses later. Therefore, it’s likely that the cause of death was due to an imbalanced immune response resulting from a prolonged and profuse viral burden [46]. Indeed, nsp6 is known to activate the NLRP3 inflammasome by suppressing acidification of lysosomes leading to pyroptosis; whether ΔSGF affects this pathway is unclear [47,48]. Furthermore, ubiquitinated nsp6 binds to transforming growth factor β-activated kinase 1 (TAK1) to activate the NF-κB signaling cascade, a major component of cytokine storms [49]. In contrast to our results, a prototype SARS-CoV-2 (with a Spike D614G mutation) bearing both BA.1 spike and nsp6 genes was attenuated [50]. Combined with our results, the collective data suggest that mutant nsp6 alone augments virulence (this study), whereas an epistatic Spike/nsp6 interaction may drive attenuation of the Omicron variant [50].

Since the beginning of the COVID-19 pandemic, SARS-CoV-2 has continued to evolve and adapt to the human host. This study demonstrates the enhanced functions of SARS-CoV-2 nsp6 by a triple deletion ΔSGF: (i) ΔSGF reduces SARS-CoV-2 susceptibility to IFN-α treatment by improving suppression of phosphorylation of STAT1 and STAT2 in the IFN-α signaling pathway; (ii) ΔSGF improves viral fitness in primary human airway cultures, as well as increases virulence in mice, likely through increased cytokine expression, leading to cytokine storm. Our study provides another example that mutations outside of structural proteins contribute to viral fitness and pathogenesis, underscoring a need to investigate the impact of novel mutations in emerging variants.

## Supplementary Material

Supplemental Material
