# Supplementary material for: Mutations in SARS-CoV-2 variant nsp6 enhance type-I interferon antagonism
Source: Emerg Microbes Infect. 2023 May 14;12(1):2209208. doi: 10.1080/22221751.2023.2209208 (PMC10184609; doi:10.1080/22221751.2023.2209208)
Supplement: Supplemental Material [file TEMI_A_2209208_SM3259.zip › Revised_nsp6 paper_Supplementary Materials.docx]

# Supplementary Materials

**Figure S1:** **ΔSGF-WA1 does not cause more severe pathology in mouse lung tissues**

Mouse lung tissue samples stained with H&E for analysis from mock, WA1-, or ΔSGF-WA1-infected mice at 4 and 6 dpi. Slight hemorrhaging in mock samples is attributable to the method of euthanasia. By 4 dpi, both WA1 and ΔSGF-WA1 caused interstitial pneumonia, perivasculitis, peribronchiolitis, and arterial mononuclear margination. By 6 dpi, alveolar edema was common.

**Table S1**

| PANGO | ΔSGF  (ORF1a:DEL3675/3677) | ΔLSG  (ORF1a:DEL3674/3676) |
| --- | --- | --- |
| B.1.1.7 | 97% | <0.5% |
| B.1.351 | 93% | <0.5% |
| P.1 | 82% | <0.5% |
| B.1.617.2 | <0.5% | <0.5% |
| BA.1 | <0.5% | 95% |
| BA.2 | 96% | <0.5% |
| BA.2.75 | 73% | <0.5% |
| BA.4 | 95% | <0.5% |
| BA.5 | 97% | <0.5% |
| B.1.1.529 | 10% | 49% |
| XBB.1.5 | 99% | <0.5% |
| BQ.1.1 | 97% | <0.5% |
| CH.1.1 | 97% | <0.5% |

**Table S2**

Primers:

| 2019-nCoV_N2-F | (5’-TTACAAACATTGGCCGCAAA-3’) |
| --- | --- |
| 2019-nCoV_N2-R | (5’-GCGCGACATTCCGAAGAA-3’) |
| HuGAPDH-F | (5’-TGTTGCCATCAATGACCCCTT-3’) |
| HuGAPDH-R | (5’-CTCCACGACGTACTCAGCG-3’) |
| Mu_GAPDH-F | (5’-AGGTCGGTGTGAACGGATTTG-3’) |
| Mu_GAPDH-R | (5’TGTAGACCATGTAGTTGAGGTCA-3’) |
| Mu_IFITM1-F | (5’-GCCACCACAATCAACATGCCTG-3’) |
| Mu_IFITM1-R | (5’-ACCCACCATCTTCCTGTCCCTA-3’) |
| Mu_ISG56-F | (5’-TACAGGCTGGAGTGTGCTGAGA-3’) |
| Mu_ISG56-R | (5’-CTCCACTTTCAGAGCCTTCGCA-3’) |
| Mu_IL-1b-F | (5’-TCGGACCCATATGAGCTGA-3’) |
| Mu_IL-1b-R | (5’-CCACAGGTATTTTGTCGTTGC-3’) |
| nsp6-F | (5’-ACCTTCTCTTGCCACTG-3’) |
| nsp6-R | (5’- AAACGAGTGTCAAGACATTCATAA -3’) |
