# Supplementary figures and images for: Mutations in SARS-CoV-2 variant nsp6 enhance type-I interferon antagonism
Source: Emerg Microbes Infect. 2023 May 14;12(1):2209208. doi: 10.1080/22221751.2023.2209208 (PMC10184609; doi:10.1080/22221751.2023.2209208)

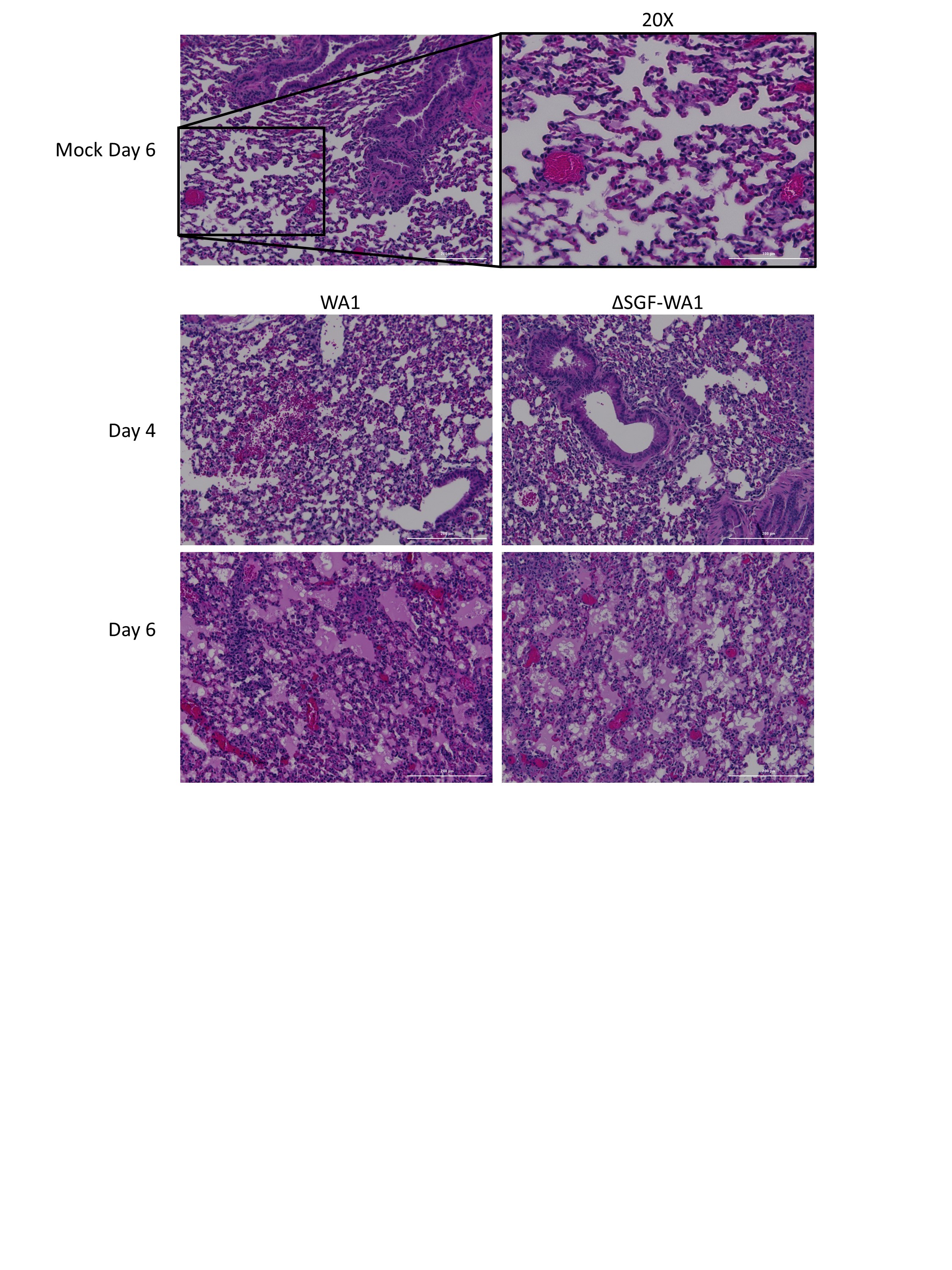

Supplement: Supplemental Material [file TEMI_A_2209208_SM3259.zip › Slide4.JPG]
